# Plant community responses to climate change in the Zayandeh-Rud Basin, Iran: Does woody plant encroachment follow global patterns?

**DOI:** 10.1186/s12862-026-02575-z

**Published:** 2026-08-17

**Authors:** Mahbobeh Hadinejad, Ali Asghar Naghipour, Ataollah Ebrahimi, Mostafa Tarkesh Esfahani, Babak Naimi

**Affiliations:** 1 Department of Nature Engineering, Faculty of Natural Resources and Earth Sciences, Shahrekord University, Shahrekord, 8818634141 Iran; 2 Department of Natural Resources, Isfahan University of Technology, Isfahan, 8415683111 Iran; 3 Quantitative Biodiversity Dynamics (QBD), Department of Biology, University of Utrecht, Padualaan 8, Utrecht, 3584 CH The Netherlands

**Keywords:** SDMs, Arid and semi arid ecosystems, Ensemble modeling, Habitat suitability

## Abstract

Arid and semi-arid ecosystems are highly sensitive to climate warming and drying, yet community-level responses in the Middle East remain poorly quantified. This study assesses the potential impacts of climate change on plant community distributions in the Zayandeh-Rud basin, central Iran, and evaluates whether woody plant encroachment follows global patterns. Species distribution models were applied to project current and future (2050 and 2070) distributions of major vegetation communities, including Semi Steppe–Shrub, Semi Steppe–Perennial Herbaceous, Alpine Vegetation, Semi Desert–Salty Plants, Semi Desert–Shrub, Semi Steppe–Semi Shrub, and Steppe–Semi Shrub, under three climate scenarios (SSP1-2.6, SSP2-4.5, and SSP5-8.5). Annual precipitation (Bio12) was identified as the primary driver shaping Semi Steppe–Shrub, Semi Steppe–Perennial Herbaceous, Semi Steppe–Semi Shrub, and Steppe–Semi Shrub communities, whereas maximum temperature of the warmest month (Bio5) and slope were influential predictors for Alpine Vegetation and semi-desert communities. In the study area, the mean (± SD) of GCMs and SSPs for the net change percentage of total woody plant communities (including Semi Steppe–Shrub, Semi Steppe–Semi Shrub, Steppe–Semi Shrub, and Semi Desert–Shrub) during the period 2050 to 2070 (over the next 60 years) was estimated at 26.86 ± 12.05%. The annual expansion rate of these woody communities was estimated at 0.45 ± 0.20%. These patterns suggest a potential trend of woody plant encroachment that may be associated with increasing temperatures and declining precipitation. Such structural changes may lead to increased dominance of woody species, shifts in community composition, biotic homogenization, and potential impacts on ecosystem functions, including carbon sequestration, nutrient cycling, soil stability, and biodiversity conservation.

**Supplementary Information:**

The online version contains supplementary material available at 10.1186/s12862-026-02575-z.

## Introduction

Climate change is widely recognized as one of the most serious environmental challenges facing human livelihoods and natural ecosystems [58, 109], with profound impacts on water resources, biodiversity and ecosystem services. Projections under high-emission scenarios suggest that the global mean temperature could increase by around 4 °C by the end of the 21st century, along with substantial shifts in precipitation regimes and an increased frequency and intensity of climate extremes [52]. These changes are likely to exacerbate hydrological imbalances and intensify ecological stress across the globe. Arid and semi-arid regions are expected to be particularly vulnerable because even small changes in temperature and rainfall can strongly alter water availability, soil moisture and vegetation dynamics.

The Middle East and North Africa region lies at the core of these projected changes and is often identified as a climate change “hotspot”, with strong warming and, in many areas, declining precipitation [63]. Iran is located at the central part of this region, already experiences chronic water scarcity, and nearly half of Iran is classified as arid or semi-arid [56, 92]. Climate projections for Iran indicate further warming on the order of 1–4.7 °C by the late 21st century and a reduction of annual precipitation, implying increased drought risk and intensifying pressure on ecosystems and livelihoods [63]. These trends threaten Iran’s exceptional floristic richness, which comprises approximately 8,000 to 9,000 plant species, many of which are endemic [85]. The Zayandeh-Rud basin, located in central Iran, is one of the largest and most socio-economically and ecologically important basins, recognized as one of the world’s major biodiversity hotspots [76, 77]. The basin spans steep gradients in elevation, temperature and precipitation, from high mountainous headwaters to low-lying semi desert plains [17], giving rise to a broad range of plant communities from the Irano-Turanian floristic region, including Semi Steppe–Shrub, Semi steppe–semi-shrub, Semi Steppe–Perennial Herbaceous, Steppe-semi shrub, Alpine vegetation, Semi Desert-Salty Plants and Semi Desert–Shrub communities. This environmental and floristic diversity underpins important ecosystem services such as forage production, soil and water conservation, carbon storage and habitat provision for numerous endemic and rare species. However, rapid warming, reduced river discharge, land use change, and unsustainable exploitation of water resources have collectively accelerated ecosystem degradation and eroded ecosystem resilience [47, 88, 115].

One of the key ecological processes linked to climate change and land-use alteration in drylands is woody plant encroachment, defined as the expansion and increased dominance of woody species at the expense of herbaceous vegetation, particularly perennial grasses [14, 15, 25, 26, 97, 104]. Woody encroachment in many arid and semi-arid regions worldwide is driven by multiple interacting factors, including rising temperatures, increasing atmospheric CO₂, shifts in precipitation patterns, overgrazing, fire, and changes in soil properties [102]. This process can substantially modify ecosystem structure and function, affecting biodiversity, primary productivity, carbon and nutrient cycling, hydrological processes, and the provision of forage for pastoral systems [15, 37].

In ecological literature, woody plant encroachment encompasses two distinct conceptual dimensions. First, it is defined as a site-level process in which the density or cover of woody species increases per unit area within an existing plant community. This form of encroachment primarily occurs in response to changes in ecological regimes, including livestock grazing, fire, precipitation variability, and rising atmospheric CO₂ levels, and has been widely documented across arid and semi-arid ecosystems. In the arid and semi-arid ecosystems of Iran, multiple lines of evidence have shown that the interaction between livestock grazing and fire can alter vegetation structure and composition [53, 99, 114]. Furthermore, studies conducted in semi-steppe rangelands of Iran indicate that grazing intensity significantly reduces herbaceous species while promoting more resistant and shrubby species [95]. In addition, environmental factors such as precipitation, soil properties, and microhabitat heterogeneity play a crucial role in controlling the establishment and expansion patterns of woody species in Iran’s dry ecosystems. Fire is also a powerful ecological driver that reshapes plant community composition and diversity. Post-fire environmental filters typically favor species adapted to warmer, drier, and more open conditions, a phenomenon referred to as community thermophilization. For example, in subalpine forests of Tibet, severe fire has been associated with increased alpha diversity and decreased beta diversity, indicating a shift toward more thermophilic species at lower elevations [60]. This mechanistic understanding of fire-driven community changes is conceptually linked to processes of habitat degradation and community reassembly in response to ecological disturbances.

In contrast, the second dimension of woody plant encroachment relates to shifts in the distribution ranges of woody plant communities in response to climate change. This process occurs at broader spatial scales and leads to changes in the climatically suitable range of these communities. Species Distribution Models (SDMs), which are based on correlative relationships between species occurrences and environmental variables, primarily assess this type of change. They are capable of predicting potential shifts in distribution ranges under future climate scenarios, rather than actual changes in local density or cover [38, 44, 64]. Therefore, the outputs of these models should be interpreted as indicators of changes in climatic suitability and potential distributional shifts of plant communities. Although woody encroachment has been documented in several dryland regions globally, the extent to which plant communities in Iranian basins such as Zayandeh-Rud follow these global patterns remains poorly understood.

Robust projections of future vegetation dynamics require quantitative tools that can link the distribution of plant communities to underlying environmental gradients and then extrapolate under climate change scenarios. Species Distribution Models (SDMs) have become widely used for this purpose, allowing researchers to relate species or communities to climatic and topographic predictors and to assess the response of plant communities to the changes in environmental conditions and project potential range shifts under alternative future climate change scenarios [13, 35]. Ensemble modeling approach, which combines multiple algorithms into a consensus prediction, generally provides higher accuracy and more robust forecasts than single algorithms by reducing model-based uncertainty [90]. The present study is the immediate predecessor of our recent work Hadinejad et al. [47], in which the distribution of plant communities in the Zayandeh-Rud basin was projected using only a single MaxEnt model, two SSP scenarios (SSP2-4.5 and SSP5-8.5), and the 2070 time horizon. In the current study, that initial framework has been substantially developed and upgraded. The present study extends our previous work in several important ways: First, the single MaxEnt model has been replaced with an ensemble of eight machine-learning algorithms to reduce model-based uncertainty; second, SSP1-2.6 has been added to bracket the lower end of emission pathways; third, the 2050 time window has been added in addition to 2070; fourth, the predictor set has been revised, in particular replacing DEM, Bio3, and Bio16 with topographic (slope, aspect), land-use, and bioclimatic (Bio5, Bio14) variables; fifth, TSS has been added alongside AUC; and sixth, the results have been embedded in the global woody-encroachment framework, which constitutes the fourth objective of this study and represents the principal conceptual contribution beyond the 2025 paper. These enhancements not only increase the accuracy and reliability of the projections but also enable an assessment of whether the patterns of change in this basin are consistent with global trends.

SDMs have been widely applied to species-level distribution modeling, but community-level modeling across multiple plant communities along strong environmental gradients remains limited, especially within Iran [18]. In this study, we apply an ensemble of machine-learning modeling approaches to assess potential responses of plant communities in the Zayandeh-Rud basin to future climate change. Specifically, our objectives are to: (1) model the current spatial distribution of seven dominant plant communities in relation to key climatic and topographic variables; (2) identify the principal environmental drivers influencing the distribution of these communities; (3) project changes in the extent and spatial patterns of plant communities under mid-century (2050) and late-century (2070) climate scenarios based on SSP1-2.6, SSP2-4.5, and SSP5-8.5; and (4) compare projected changes in the distribution and suitable habitat of different woody plant communities under future climate scenarios to determine whether their responses are uniform or community-specific, and whether they collectively support the globally reported pattern of woody plant encroachment in dryland ecosystems.

Through these objectives, this study provides insights into vegetation dynamics in a climatically sensitive basin and offers information relevant to biodiversity conservation and climate-adapted land management in arid and semi-arid regions.

## Materials and methods

### Study area

**Fig. 1:**
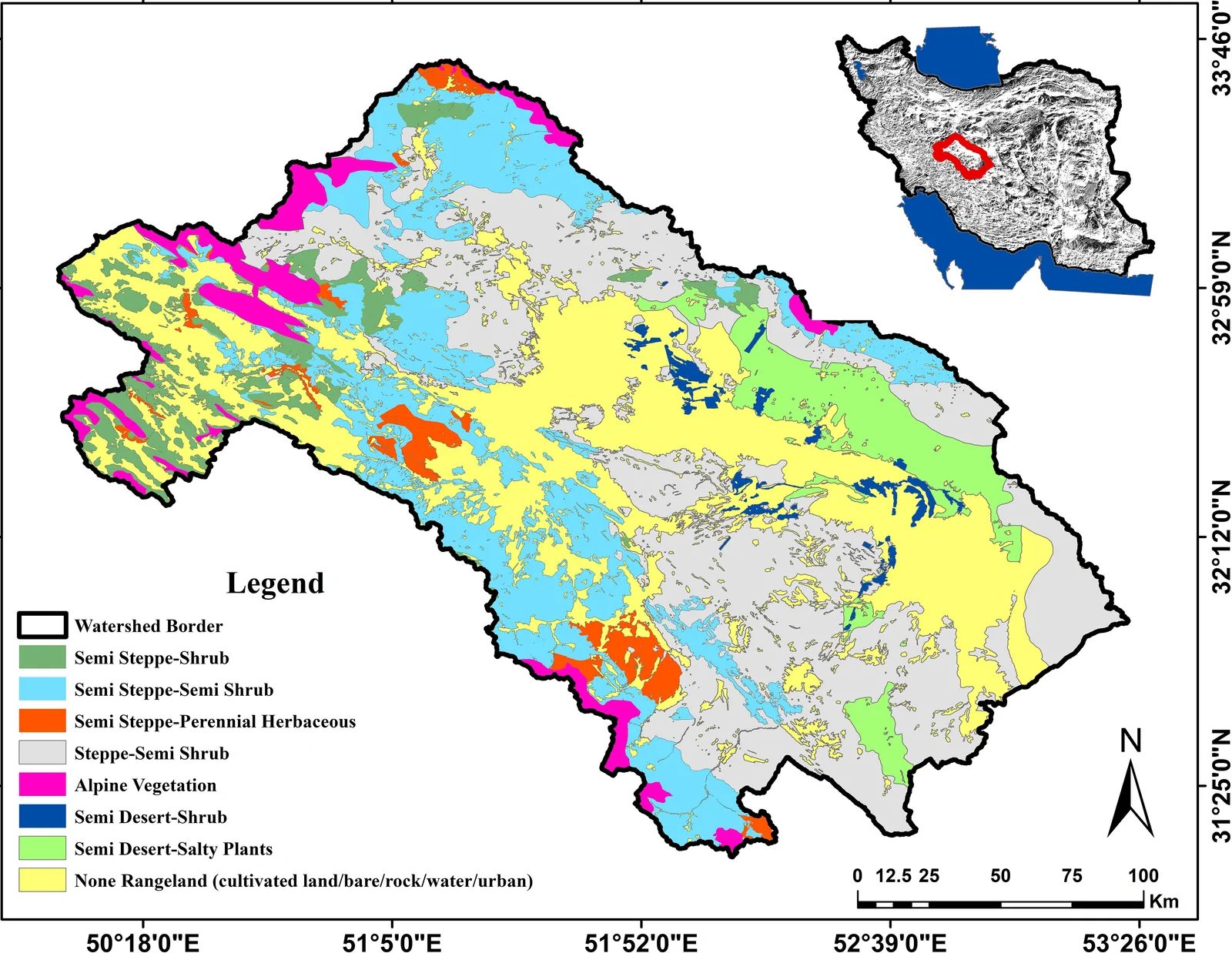
Location map of plant communities in the Zayandeh-Rud basin, Iran

The Zayandeh-Rud basin, with an area exceeding 41,529.94 km², represents one of the largest drainage basins in the Central Iranian Plateau. The basin is located between 31°25′–33°46′N latitude and 50°18′–53°26′E longitude (Fig. 1). It spans several provinces, including Isfahan, Chaharmahal-va-Bakhtiari, Yazd, and Fars. The Zayandeh-Rud River originates in the central Zagros Mountains, particularly from Zardkuh in Chaharmahal-va-Bakhtiari Province, and flows approximately 350–400 km eastward across the central Iranian plateau, ultimately discharging into the Gavkhuni Wetland [68].

Climatically, the basin lies within an arid to semi-arid zone. Due to its geographical extent, climatic variability, and geological significance, it serves as a primary framework for hydrological, environmental, and water resources management studies in the region [117]. Climate change has exerted significant impacts on the Zayandeh-Rud basin, and multiple studies and projections indicate that the region will experience decreasing precipitation and rising temperatures in the future [46].

### Vegetation data

In the present study, the vegetation type map prepared in 2020 by the Natural Resources and Watershed Management Organization of Iran was used as the primary basis for identifying vegetation units. The vegetation types delineated in these maps represent relatively homogeneous areas in terms of species composition, dominant species, and habitat conditions, and therefore provide an appropriate starting point for the delineation of plant communities. Based on these maps, vegetation units within the study area were identified, and information on dominant and accompanying species, as well as general habitat characteristics of each type, was extracted to obtain an initial understanding of the ecological structure of the region.

Subsequently, the characteristics of each vegetation type were evaluated from a phytosociological perspective. Specifically, species composition, dominance patterns, habitat homogeneity, and the repeatability of vegetation structure within each type were analyzed to determine which units truly represent distinct plant communities in terms of ecological structure and function. This approach allows for distinguishing between vegetation types that may differ only nominally or descriptively but belong to the same ecological community, and those that exhibit distinct ecological characteristics and should therefore be considered separate communities.

To ensure the reliability of this classification, field verification was conducted across representative sampling locations within each vegetation type. Field observations included recording dominant and associated species, assessing habitat conditions, evaluating vegetation homogeneity or heterogeneity, and comparing field data with map-based information. This step contributed to refining community boundaries and correcting potential inconsistencies between mapped units and actual field conditions.

As a result, vegetation types with similar species composition and structural characteristics were aggregated into a single plant community, whereas those exhibiting distinct dominance patterns or ecological conditions were classified as separate communities. Ultimately, this process led to the transformation of the initial vegetation type map into a more refined map of actual plant communities in the study area. This updated map, grounded in both official data and phytosociological principles and validated through field observations, provides a robust basis for subsequent modeling of plant community distributions.

Finally, based on the refined plant community map, the habitat classification proposed by Pabo [79], and the classification framework of Feyzi and Shirani [40], the vegetation of the study area was categorized into seven main plant communities, as presented in Table 1.

In this study, plant communities were classified and named based on two main criteria. First, climatic conditions, particularly precipitation and temperature (Table 1), were used as the primary basis for classification. Second, the morphological characteristics (life forms) of the dominant species within each community were considered (Fig. S1).

**Table 1:** Plant communities of the Zayandeh-Rud basin [47]

Vegetation type	Description
Semi Steppe: Includes: 1- Semi Steppe-Shrub, 2- Semi Steppe-Semi Shrub and 3- Semi Steppe-Perennial Herbaceous plant communities	The annual precipitation in the semi-steppe region varies between 230 and 400 millimeters. The flora of this region is richer than that of the steppe region, and various shrub, semi-shrub, and perennial herbaceous species are observed in this area.
1- Semi Steppe-Shrub	The dominant plant species in this region are shrubs, including *Astragalus verus* Olivier, *Astragalus brachycalyx* Fisch., and *Astragalus susianus* Boiss. A total of 346 occurrence points were recorded for this plant community.
2- Semi Steppe-Semi Shrub	The majority of the dominant plant species in this region are semi-shrubs, including *Artemisia aucheri* Boiss. and *Scariola orientalis* (Boiss.) Soják. A total of 115 occurrence points were recorded for this plant community.
3- Semi Steppe-Perennial Herbaceous	The dominant plant species in this region are perennial herbaceous species, including *Stipa hohenackeriana* Trin. & Rupr., *Bromus tomentellus* Boiss., and *Agropyron* sp. A total of 45 occurrence points were recorded for this plant community.
Steppe: Includes: 4- Steppe-Semi Shrub	The annual precipitation in the steppe region ranges between 100 and 230 millimeters. Various shrub and semi-shrub plant species are found in this region.
4- Steppe-Semi Shrub	*Artemisia sieberi* Besser characterizes the flora of this region. *Anabasis aphylla* L. is another dominant species in this area [79]. A total of 112 occurrence points were recorded for this plant community.
5- Alpine Vegetation	In this study, elevations above 2,700 m were considered as the high mountain region. Various species exhibit significant growth and expansion in this area. The dominant plant species in this region include *Amygdalus* spp., *Acantholimon* sp., *Acanthophyllum* sp., and *Astragalus* spp. A total of 77 occurrence points were recorded for this plant community.
Semi Desert: Includes: 6- Semi Desert-Salty Plants and 7- Semi Desert-Shrub plant communities	The annual precipitation in this region is less than 100 millimeters. The average temperature in January ranges from 4 to 10 °C, while the average temperature in July ranges from 29 to 34 °C.
6- Semi Desert-Salty Plants	The majority of plant species in this region consist of salt-tolerant species, such as *Halocnemum strobilaceum* (Pall.) M.Bieb, and *Seidlitzia rosmarinus* Boiss. Pabo, [79]. A total of 100 occurrence points were recorded for this plant community.
7- Semi Desert-Shrub	The majority of plant species in this region consist of *Haloxylon aphyllum* (Minkw.) Iljin and *Haloxylon persicum* Bunge A total of 46 occurrence points were recorded for this plant community.

### Environmental data

Environmental variables comprised a land use map prepared by the Natural Resources and Watershed Management Organization of the country, along with a digital elevation model (DEM) and bioclimatic variables obtained from the WorldClim database. Terrain-derived variables, including slope and aspect, were extracted from the DEM within a Geographic Information System (GIS) framework and incorporated as physiographic predictors.

Nineteen bioclimatic variables were obtained at a spatial resolution of 30 arc-seconds (~ 1 km²) from the WorldClim v2.1 database for the baseline period [41]. For future climate projections, data from two General Circulation Models (GCMs), MRI-ESM2-0 and HadGEM3-GC31-LL, were used [2, 113] under three Shared Socioeconomic Pathways (SSPs 1–2.6, 2–4.5, and 5–8.5) for two time periods: 2041–2060 (2050) and 2061–2080 (2070). Previous studies have demonstrated that these two GCMs perform reliably in simulating climate over Iran [2, 113].

To reduce collinearity, correlations among bioclimatic and physiographic variables were assessed using Pearson’s correlation coefficients in SPSS. Variables with *r* > 0.8 were excluded [34]. Hidden multicollinearity was further tested in R using the usdm package [71]. Principal Component Analysis (PCA) was first applied to identify key contributing variables, and Variance Inflation Factor (VIF) values were calculated. Variables with VIF > 5 were considered collinear and removed [57] (Tables S1 and S2).

Ten relatively independent environmental variables were ultimately retained for modeling: land use, aspect, slope, mean diurnal range (Bio2), temperature seasonality (Bio4), maximum temperature of the warmest month (Bio5), temperature annual range (Bio7), annual total precipitation (Bio12), precipitation of the driest month (Bio14), and precipitation seasonality (Bio15). It should be noted that slope, aspect, and land use were considered constant in both the current and future modeling.

### Modeling the distribution of plant communities

For modeling each plant community, presence points of each community (spaced 1 km apart) along with the selected environmental variables were used as inputs to the modeling process.

Species Distribution Models (SDMs) were developed separately for each plant community using seven commonly applied machine learning algorithms implemented in the sdm package in R [72]. These algorithms included the Generalized Linear Model (GLM; [65]), Classification and Regression Tree (CART; [23]), Boosted Regression Tree (BRT; [42]), Multivariate Adaptive Regression Splines (MARS; [43]), Random Forest (RF; [24]), Support Vector Machine (SVM; [103]), Maximum Entropy (MaxEnt; [83]) and Recursive Partitioning and Regression Trees (RPART; [100]).

A total of 1,000 background points were randomly generated within the study area and used as pseudo-absence data together with presence points for model training. Model performance was assessed using the bootstrap resampling method with five replicates, which randomly partitions the dataset into training and testing subsets [73].

Model performance was evaluated using the Area Under the Receiver Operating Characteristic Curve (AUC) and the True Skill Statistic (TSS). AUC values range from 0.5 (no discrimination) to 1 (perfect discrimination), while TSS values range from − 1 to 1, with higher values indicating greater predictive accuracy [31].

For each plant community, 40 individual models (8 algorithms × 5 replicates) were generated and combined into an ensemble prediction [36, 72]. Each model’s contribution to the ensemble was weighted according to its AUC value. This ensemble approach enhances predictive accuracy and accounts for model uncertainty [12].

Modeling was performed for the current period (1970–2000) and for 2050 and 2070 under three SSP scenarios (SSP1–2.6, SSP2–4.5, and SSP5–8.5) using the two GCMs described above. Habitat suitability maps were classified into suitable and unsuitable categories based on the optimal TSS threshold (Table S3). Areas that became newly suitable were defined as Gain, whereas areas that became unsuitable were defined as Loss. The spatial extent of Gain and Loss for each plant community was estimated for 2050 and 2070 under all climate scenarios.

All modeling procedures were conducted following the ODMAP v1.0 reporting protocol [121], and the completed protocol table is provided as Supplementary Table S4.

## Results

### Model performance and variable importance

The ensemble values of the Area Under the Curve (AUC) and the maximum True Skill Statistic (max-TSS) were obtained for each plant community in the study area (Table 2 and S5). The highest AUC value was related to the Alpine Vegetation community (AUC = 0.99), while the lowest AUC was observed for the Steppe–Semi Shrub community (AUC = 0.91). Furthermore, the maximum TSS value belonged to the Semi Steppe–Perennial Herbaceous community (TSS = 0.94), and the minimum TSS value was recorded for the Steppe–Semi Shrub community (TSS = 0.70).

**Fig. 2:**
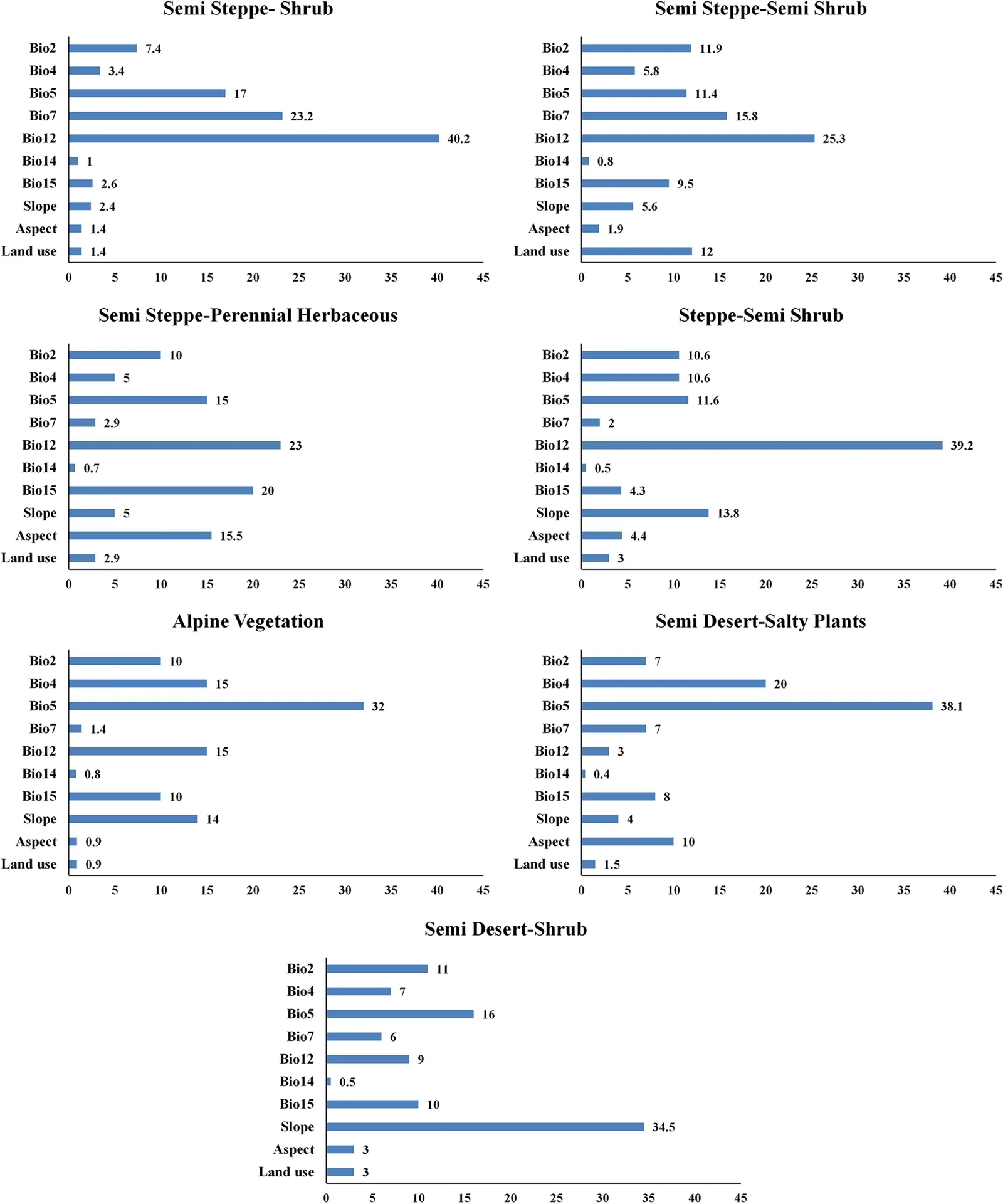
Relative importance of environmental predictors in ensemble SDMs for plant communities of the Zayandeh-Rud basin, Iran

In total, ten environmental variables were used to model the seven plant communities. The relative importance of these variables varied among communities (Fig. 2). Annual precipitation (Bio12) was identified as the most influential variable for the Semi Steppe–Shrub, Semi Steppe–Semi Shrub, Semi Steppe–Perennial Herbaceous, and Steppe–Semi Shrub communities. In contrast, the maximum temperature of the warmest month (Bio5) and slope were the most important predictors for the Alpine Vegetation, Semi Desert–Salty Plants, and Semi Desert–Shrub communities, respectively (Fig. 2).

**Table 2:** Ensemble model validation results

Plant Communities	AUC	TSS	Boyce Index
Semi Steppe-Shrub	0.97	0.84	0.997
Semi Steppe-Semi Shrub	0.95	0.77	0.993
Semi Steppe-Perennial Herbaceous	0.98	0.94	0.887
Steppe-Semi Shrub	0.91	0.7	0.991
Alpine Vegetation	0.99	0.90	0.957
Semi Desert-Salty Plants	0.96	0.85	0.977
Semi Desert-Shrub	0.98	0.92	0.966

### Modeling the current distribution of plant communities in the study area

**Fig. 3:**
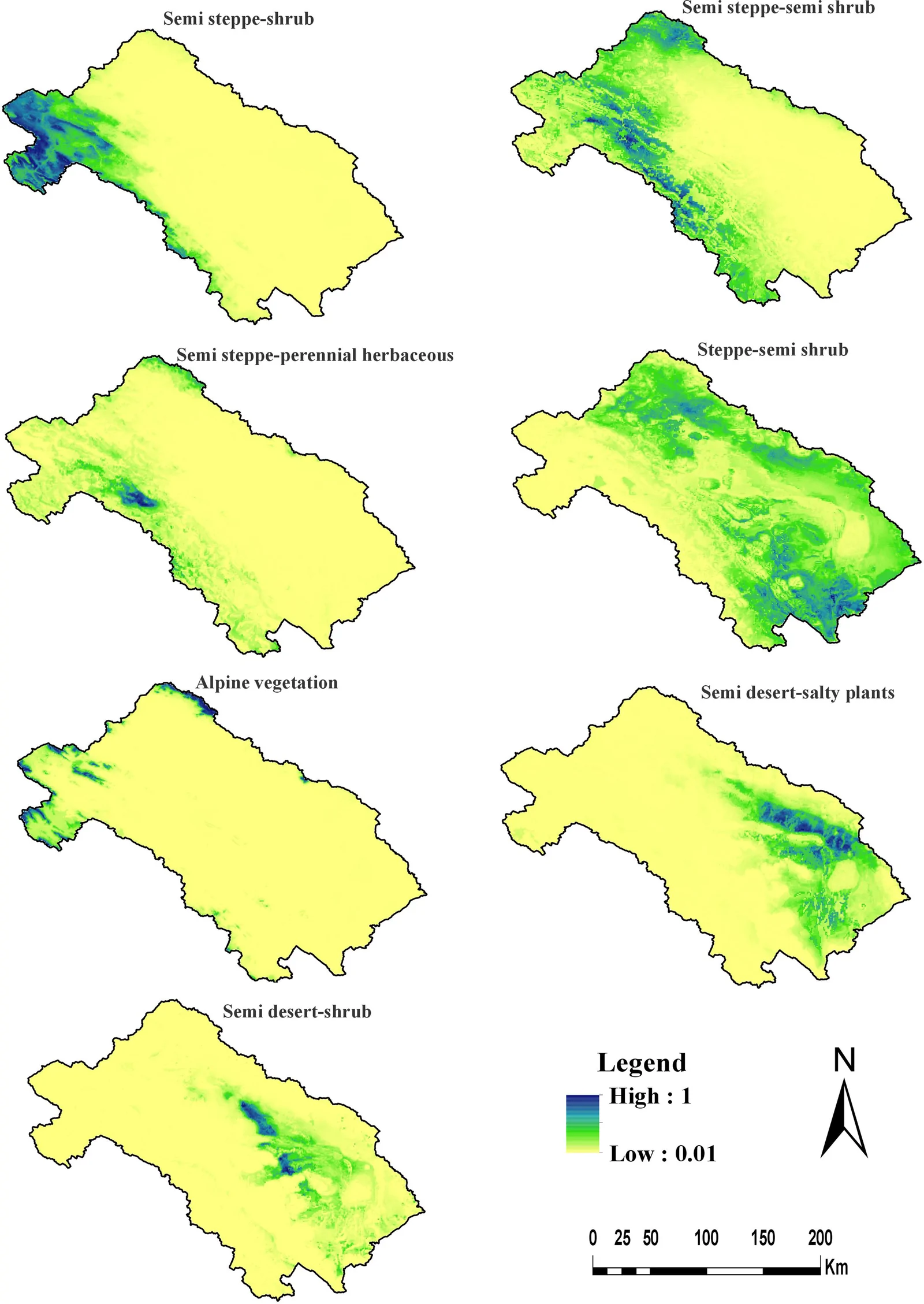
Spatial distribution of the current (1970–2000) suitable habitats for each plant community in the Zayandeh-Rud basin, Iran

The predicted current spatial distribution of plant communities is presented in Table 3; Fig. 3.

1. **Semi steppe–shrub**:

The suitable habitat of this community covers 4,644.65 km² (10.85% of the study area) and is mainly distributed in the northwestern parts of the basin.

2. **Semi steppe–semi shrub**:

This community occupies approximately 6,356.62 km² (14.84%), predominantly across the northern and northwestern regions, with limited presence in southwestern areas.

3. **Semi steppe–perennial herbaceous**:

The suitable habitat covers about 2,760 km² (6.44%) and is mainly concentrated in the western parts of the study area.

4. **Steppe–semi shrub**:

This community represents the largest suitable area, covering 18,291.81 km² (42.71%), and is widely distributed across the central and northeastern to southeastern regions, forming extensive vegetation cover.

5. **Alpine vegetation**:

The suitable habitat is estimated at 2,574.46 km² (6.01%), primarily concentrated in northwestern areas with scattered occurrences in northern parts.

6. **Semi desert–salty plants**:

This community occupies approximately 5,109.66 km² (11.93%) and is mainly distributed in eastern regions, showing relatively uniform coverage.

7. **Semi desert–shrub**:

The suitable habitat covers about 3,091.67 km² (7.22%), largely concentrated in central areas, with sparse and scattered distribution in the southeast.

**Table 3:** Estimated current suitable habitat area (km²) and corresponding percentage for each plant community

Plant Communities	Current	
	Suitable Habitat (km^2^)	Suitable Habitat (%)
Semi Steppe-Shrub	4644.65	10.85
Semi Steppe-Semi Shrub	6356.62	14.84
Semi Steppe-Perennial Herbaceous	2760.00	6.44
Steppe-Semi Shrub	18291.81	42.71
Alpine Vegetation	2574.46	6.01
Semi Desert-Salty Plants	5109.66	11.93
Semi Desert-Shrub	3091.67	7.22

### Future distribution of plant communities

Overall, projections for 2050 and 2070 under all SSP scenarios indicate that climate change will drive contrasting expansion and contraction patterns among plant communities, depending on their ecological traits and climatic tolerance (Table 4; Table S6; Fig. 4, Figs. S2-S8).

**Fig. 4:**
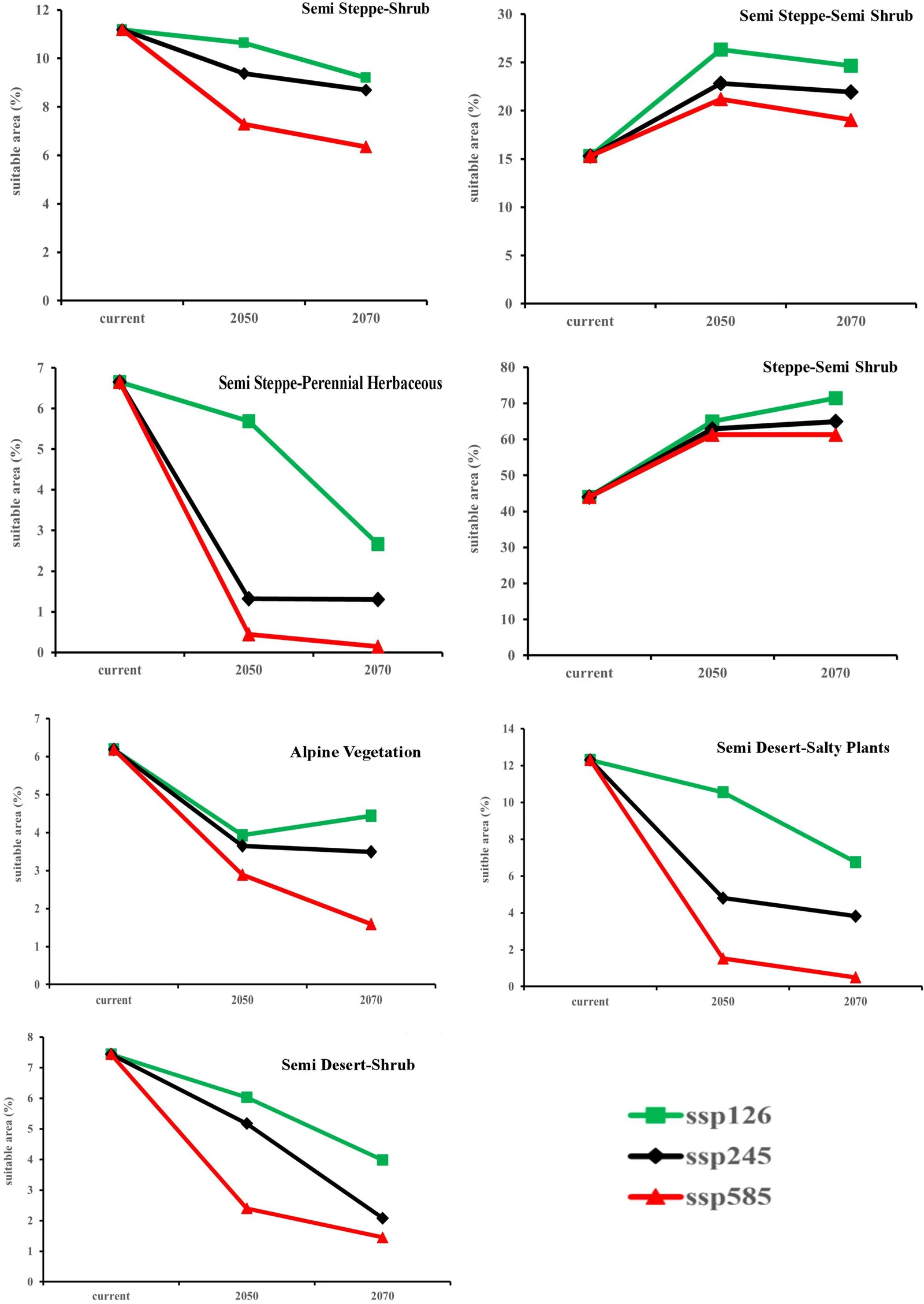
Percentage of suitable habitat area in the current period, 2050, and 2070 under three scenarios (SSP1-2.6, SSP2-4.5, and SSP5-8.5) for each plant community in the Zayandeh-Rud basin, Iran

1. **Semi steppe–shrub**:

Future projections under SSP1-2.6, SSP2-4.5, and SSP5-8.5 show a consistent reduction in suitable habitat compared to current conditions. Habitat loss intensifies by 2070, with the greatest decline under SSP5-8.5, ranging from 49.77% to 52.14% (Fig. 4, Fig. S5). These results are consistent with the mean projected net changes reported in Table 4, which indicate negative trends across all scenarios, ranging from − 4.90 ± 2.98% (SSP1-2.6, 2050s) to − 43.85 ± 1.48% (SSP5-8.5, 2070s).

2. **Semi steppe–semi shrub**:

Model projections indicate an overall increase in suitable habitat across all scenarios for 2050 and 2070, ranging from 20.35% to 39.77% (Table S6). Geographic expansion is mainly projected toward northern areas, except under SSP1-2.6 (2050) and SSP5-8.5 (2070) (Fig. S3). This increasing trend is supported by Table 4, where positive net changes are observed across all scenarios, ranging from + 18.80 ± 15.35% to + 75.39 ± 12.52%.

3. **Semi steppe–perennial herbaceous**:

All scenarios project a marked reduction and spatial shift in suitable habitats by 2050 and 2070 (Fig. S5). The extent of habitat loss ranges from 33.93% to 96.62%. Similarly, Table 4 shows consistently strong negative changes, with values ranging from − 14.37 ± 20.22% to − 97.81 ± 1.73%, confirming the high vulnerability of this community.

4. **Steppe–semi shrub**:

Suitable habitat area is projected to increase under future climate conditions, with expansion ranging from 37.66% to 66.24%, primarily toward the northwestern parts of the study area (Table S6; Fig. S5). This pattern is further supported by Table 4, which indicates positive net changes across all scenarios, ranging from + 36.06 ± 15.44% to + 62.26 ± 1.37%.

5. **Alpine vegetation**:

Projections indicate a substantial decline in suitable habitats by 2050 and 2070, with reductions ranging from 39% to 97%, mainly in northern regions (Fig. S6). According to Table 4, this decline is reflected in negative net changes across all scenarios, ranging from − 30.71 ± 18.49% to − 77.60 ± 28.43%.

6. **Semi desert–salty plants**:

Suitable habitat is projected to decline across all scenarios, with reductions ranging from 57% to 99% and a maximum loss rate of 73 km² per year. Habitat loss occurs primarily in eastern areas, with a northward shift of remaining suitable zones (Fig. S7). Table 4 also indicates substantial negative changes, ranging from − 34.36 ± 13.10% to − 92.55 ± 7.40%.

7. **Semi desert–shrub**:

Future projections indicate substantial habitat reduction and spatial shifts by 2050 and 2070 (Fig. S8), with suitable areas becoming fragmented and declining by 51% to 99% across scenarios. This is consistent with Table 4, where negative net changes range from − 18.92 ± 8.85% to − 80.46 ± 17.46%.

**Table 4:** Mean projected changes in suitable habitat across GCMs (MRI-ESM2-0 and HadGEM3-GC31-LL) and SSP scenarios (SSP1-2.6, SSP2-4.5, and SSP5-8.5) (Net Change % (Mean ± SD))

Year	Climate scenario	Plant community						
		Semi Steppe-Shrub	Semi Steppe-Semi Shrub	Semi Steppe-Perennial Herbaceous	Steppe-Semi Shrub	Alpine Vegetation	Semi Desert-Salty Plants	Semi Desert-Shrub
2050s	SSP1-2.6	-4.90 ± 2.98	+ 75.39 ± 12.52	-14.37 ± 20.22	+ 62.26 ± 1.37	-37.24 ± 9.67	-34.93 ± 44.80	-18.92 ± 8.85
	SSP2-4.5	-16.04 ± 2.67	+ 48.36 ± 3.95	-77.13 ± 0.18	+ 47.46 ± 13.37	-45.76 ± 4.46	-65.30 ± 26.79	-30.49 ± 22.81
	SSP5-8.5	-29.99 ± 19.19	+ 40.74 ± 9.50	-87.12 ± 4.83	+ 42.26 ± 15.52	-40.62 ± 3.83	-81.05 ± 13.92	-67.58 ± 7.11
2070s	SSP1-2.6	-17.67 ± 3.44	+ 57.87 ± 2.89	-59.90 ± 1.57	+ 47.55 ± 12.33	-30.71 ± 18.49	-34.36 ± 13.10	-46.39 ± 34.29
	SSP2-4.5	-26.25 ± 2.21	+ 25.29 ± 14.55	-89.36 ± 9.15	+ 42.75 ± 7.27	-44.74 ± 8.49	-64.14 ± 42.10	-71.98 ± 10.93
	SSP5-8.5	-43.85 ± 1.48	+ 18.80 ± 15.35	-97.81 ± 1.73	+ 36.06 ± 15.44	-77.60 ± 28.43	-92.55 ± 7.40	-80.46 ± 17.46

## Discussion

Modeling species distribution and ecological community structure is essential for biodiversity conservation and habitat management [74]. Predicting distribution patterns over space and time supports effective conservation planning and helps understand the links between the biodiversity crisis and climate change caused by human activities [11].

A) **Effects of environmental variables on the distribution of plant communities in the study area:**

Annual precipitation (Bio12) was identified in this study as the most influential factor determining the distribution of Semi Steppe–Shrub, Semi Steppe–Semi Shrub, Semi Steppe–Perennial Herbaceous, and Steppe–Semi Shrub communities. This finding is consistent with previous research that also highlighted the importance of Bio12 in shaping vegetation distribution across similar ecosystems [7, 48, 91]. In contrast, the maximum temperature of the warmest month (Bio5) and slope were found to significantly affect the distribution of Alpine Vegetation, Semi Desert–Salty Plants, and Semi Desert–Shrub communities. These results align with earlier studies showing that Bio5 is a key determinant of thermal tolerance, growth, reproduction, and species survival in alpine and semi desert regions, while slope and microclimatic variations further shape distribution patterns under climate change [3, 51, 61].

B) **Prediction of current and future distribution of plant communities**

Overall, the assessment of climate change impacts on the distribution of plant communities revealed that these communities do not respond uniformly to future climate scenarios, but instead exhibit distinct patterns. In Semi Steppe communities, an increase in distribution area is projected for the Semi Steppe–Semi Shrub community, whereas Semi Steppe–Shrub and Semi Steppe–Perennial herbaceous communities are expected to experience a reduction in their distribution ranges. In the steppe community (Steppe–Semi Shrub), an increasing trend in area is observed. In contrast, alpine vegetation is projected to undergo a significant reduction in its distribution range in the future. Similarly, Semi Desert communities, including Semi Desert–Salty Plants and Semi Desert–Shrub, are expected to decline in area. These findings indicate a heterogeneous response of plant communities to climate change, largely dependent on their ecological characteristics. To further elaborate on these results, a more detailed analysis of each community is presented in the following sections.

**1. Semi steppe–shrub**

Our results indicate a projected decline of the Semi Steppe–Shrub community under future climate scenarios. This community, dominated by *Astragalus verus*, *A. brachycalyx*, and *A. susianus*, is expected to lose suitable habitat due to reduced snow cover affecting snow-adapted species and increased wildfire risk driven by higher temperatures, dense vegetation, and lower humidity [8, 10, 21, 33, 48, 55, 69, 82, 91, 110]. Remaining populations persist at higher elevations, shifting upward and causing range contraction, altered species composition, and localized biodiversity loss [7, 87].

**2.** **Semi steppe–semi shrub**

The Semi Steppe–Semi Shrub community is projected to expand under future climate change, mainly toward Semi Steppe–Shrub community. Dominant species such as *A. aucheri* and *S. orientalis* benefit from rising temperatures and reduced precipitation. Within a trait-based ecological framework, *A. aucheri* is a characteristic shrub of mountainous arid and semi-arid environments, exhibiting a high ecological tolerance to combined temperature and moisture stress [54]. Such adaptations include efficient root systems for exploiting limited soil moisture and conservative water-use strategies commonly reported for species within the genus *Artemisia* [19, 30].

*S. orientalis* exhibits high drought and heat tolerance, physiological plasticity, and colonization capacity in low-competition environments such as abandoned lands [5, 89]. However, its rapid expansion raises concerns regarding invasive potential, emphasizing the need for monitoring and management [6, 70].

In the case of *S. orientalis*, in addition to its colonization ability, this species exhibits key morphological and phenological drought-adaptive traits, including reduced leaf area, thick cuticles, and early seasonal leaf shedding. This drought-avoidance strategy reduces transpiration and water loss, enabling persistence during extended dry periods [89].

**3. Semi steppe–perennial herbaceous**

This community is projected to decline under future climate scenarios. Dominant species (*Stipa hohenackeriana*, *Bromus tomentellus*, *Agropyron* spp.) are highly sensitive to warming and drying due to shallow root systems [39, 98]. Increased evapotranspiration, reduced rainfall, and phenological mismatches shorten growing seasons and reduce productivity [84]. Although elevated CO₂ may slightly improve water-use efficiency, its mitigating effect is limited under severe drought and heat stress [62, 78, 101]. Consequently, woody species with deeper roots and higher thermal tolerance gain a competitive advantage, accelerating herbaceous decline [4, 32, 66, 94].

**4. Steppe–semi shrub**

The Steppe–Semi Shrub community is expected to expand under future climate conditions. Increasing aridity favors drought-tolerant woody species with deep roots, high water-use efficiency, and protective leaf traits [80, 107]. Competition occurs both between woody and herbaceous species and among woody taxa, with climate-sensitive species such as Astragalus spp. likely replaced by more tolerant steppe species. Similar compositional shifts driven by soil moisture decline have been documented in China [106, 119, 120].

The dominant species *Artemisia sieberi* exhibits strong physiological adaptations to combined heat and drought stress, including osmolyte accumulation, antioxidant activity, and carbohydrate reallocation [9, 50, 116]. This species is a key component of lowland and mid-elevation arid and semi arid ecosystems in the Irano-Turanian region, characterized by a broad ecological amplitude across soil types and climatic gradients [67]. Its distribution is strongly influenced by soil properties such as texture, lime content, and moisture availability, emphasizing the importance of belowground traits in water acquisition.

At the individual level, *A. sieberi* responds to drought stress through reduced stomatal conductance and transpiration rate, along with osmotic adjustment via the accumulation of compatible solutes such as proline and soluble sugars [1]. In addition, the development of deep root systems and increased root-to-shoot ratios enhances access to deeper soil moisture and improves water uptake efficiency [19, 30].

**5. Alpine vegetation**

Alpine vegetation is projected to decline under future climate scenarios. Dominant species (*Amygdalus*, *Acantholimon*, *Acanthophyllum*, *Astragalus* spp.) are highly vulnerable due to narrow climatic tolerances and shallow soils. Rising temperatures increase evapotranspiration and drive upward species migration, resulting in habitat loss and summit traps [86, 105]. Thermophilization, replacement by fast-growing species, and reduced adaptive capacity further exacerbate decline [59, 75]. Extreme events, earlier snowmelt, and reduced snow cover intensify drought stress and mortality [96, 105].

**6. Semi desert–salty plants**

This community, dominated by *Halocnemum strobilaceum*, and *Seidlitzia rosmarinus*, is expected to decline under future climate change. Rising temperatures and reduced precipitation increase soil salinity and aridity, limiting growth, photosynthesis, and regeneration of halophytes [27, 28, 49, 93]. Recurrent droughts and heatwaves cause widespread shrub mortality, a trend consistent with global observations in China and Namibia [22, 112].

**7. Semi desert–shrub**

The Semi Desert–Shrub community, dominated by *Haloxylon aphyllum* and *H. persicum*, is projected to decline under future climate scenarios. Although these species exhibit strong drought tolerance, the pace of climate change exceeds their adaptive capacity, leading to metabolic stress, canopy dieback, and reduced regeneration [81, 108, 111]. Low seedling survival under repeated droughts results in population aging, while limited dispersal restricts migration to newly suitable habitats [16, 29, 118]. Without effective management of water resources, grazing pressure, and habitat connectivity, the stability of Iran’s semi-arid ecosystems may be severely compromised.

### Are the projected changes consistent with global woody-encroachment patterns?

Drivers of woody plant encroachment include climate change, increasing atmospheric CO₂, herbivory, fire, and human disturbances such as land-use change [15, 45]. Venter et al. [104] reported that, in Africa, factors other than CO₂ account for approximately 78% of woody encroachment. The magnitude and rate of woody cover expansion vary across regions worldwide, largely depending on climatic drivers and management practices. For example, the highest rates have been reported in South America and the lowest in Australia, which have been attributed to differences in fire regimes, CO₂ enrichment, grazing pressure, and soil and vegetation characteristics [97].

In the present study area, the mean (± SD) of GCMs and SSPs for the net change percentage of total woody plant communities (including Semi Steppe–Shrub, Semi Steppe–Semi Shrub, Steppe–Semi Shrub, and Semi Desert–Shrub) over the period 2050 to 2070 (i.e., over the next 60 years) was estimated at 26.86 ± 12.05%, and the annual expansion rate of these woody communities was estimated at 0.20 ± 0.45%.

Globally, absolute rates of woody plant expansion range from 0 to 3.3% cover per year, with an average of 0.85% per year [15]. According to [104]; woody cover in sub-Saharan Africa has increased by approximately 8% over the past three decades. In North America, woody encroachment rates vary among ecoregions, ranging from 0.1 to 2.3% cover increase per year [20]. Similarly, reported rates across savannas and forest–savanna boundaries in Africa, Australia, and South America range from 0.1 to 1.1% cover increase per year [97].

It is also important to note that different woody communities exhibit heterogeneous responses, with some benefiting and others declining. The changes assessed in this study are driven primarily by climate variables, while other important drivers such as herbivory, fire, land-use change, and elevated CO₂ were not explicitly included. These additional factors can either intensify woody encroachment [104] or act as management controls.

Finally, the overall trends observed in this study are consistent with those reported in a previous study by [47]. However, minor differences in the projected areas of plant communities between the two studies can be attributed to methodological differences, including modeling algorithms, threshold selection, predictor variable sets, and variation in the number of presence records for certain communities.

## Conclusion

This study demonstrates pronounced climate-driven shifts in plant community distributions in the Zayandeh-Rud Basin. Several communities, including Semi Steppe–Shrub, Semi Steppe–Perennial Herbaceous, Alpine Vegetation, Semi Desert–Salty Plants, and Semi Desert–Shrub, are projected to lose substantial suitable habitat under future climate scenarios, while Semi Steppe–Semi Shrub and Steppe–Semi Shrub communities are expected to expand. These patterns likely indicate climate-driven biotic homogenization.

The modeled responses align with global dryland trends, where warming, declining precipitation, and reduced soil moisture favor deep-rooted, drought-tolerant woody species. In the Zayandeh-Rud Basin, precipitation, temperature extremes, and topography emerged as key drivers of community shifts, suggesting increasing spatial displacement and fragmentation under ongoing climate change.

These changes pose significant risks to biodiversity, ecosystem functioning, and land productivity, while increasing the likelihood of desertification. Proactive adaptation measures such as restoring drought-tolerant native vegetation, improving water-use efficiency, and regulating grazing are essential to enhance ecosystem resilience and support sustainable land-use planning in the basin and similar arid and semi-arid regions.

## Limitations

This study has several limitations that should be considered when interpreting the results. First, species distribution models (SDMs) rely on a number of key assumptions, including niche conservatism, absence of dispersal limitation, lack of biotic interactions, and equilibrium with current climatic conditions. These assumptions may not fully hold in real-world systems, thereby introducing uncertainty into model projections.

Second, climate-related uncertainty remains an important source of error, as only two general circulation models (GCMs) were used in this study, which may not fully capture the range of possible future climatic conditions.

Third, land-use conditions were assumed to remain constant through 2050 and 2070, whereas future land-use changes could substantially influence vegetation distribution patterns.

Fourth, important drivers such as atmospheric CO₂ increase: It should be noted that although elevated atmospheric CO_2_ concentration is widely acknowledged as a major global driver of woody encroachment, it was not explicitly included as a predictor in the present modeling framework. This is primarily due to the absence of spatially explicit CO2 datasets at a resolution comparable to the environmental variables employed in this study. Instead, the modeling approach was based on climatic and physiographic predictors, within which the influence of CO_2_ is implicitly represented through climate projections derived from general circulation models under different emission scenarios.

However, it is important to recognize that this indirect representation does not fully capture the physiological and ecological effects of CO_2_ fertilization, which may play a significant role in promoting woody plant expansion. Consequently, the exclusion of CO_2_ as an independent variable constitutes a limitation of this study. This limitation is particularly relevant when interpreting the extent to which the study area conforms to global patterns of woody encroachment. Therefore, any inference regarding the alignment of the basin with global encroachment trends should be made with caution, acknowledging that not all key global drivers have been explicitly incorporated.

Fifth, model projections may involve extrapolation into non-analogue climatic conditions. In this context, approaches such as multivariate environmental similarity surface (MESS) analysis or extrapolation detection methods would be valuable to assess the extent of extrapolation uncertainty.

Sixth, sample sizes for some communities were relatively small (e.g., Semi Steppe–Perennial Herbaceous, *n* = 45; Semi Desert–Shrub, *n* = 46), which may affect model robustness and predictive performance.

Finally, the use of random cross-validation in the presence of spatial autocorrelation may lead to inflated estimates of model performance, particularly for metrics such as AUC, and should therefore be interpreted with caution.

## Supplementary Information

Below is the link to the electronic supplementary material.

Supplementary Material 1

Supplementary Material 2

Supplementary Material 3

Supplementary Material 4

Supplementary Material 5

Supplementary Material 6

Supplementary Material 7

Supplementary Material 8

Supplementary Material 9

## Data Availability

The data that support the findings of this study are available from the corresponding author upon request.
