# Supplementary material for: Plant community responses to climate change in the Zayandeh-Rud Basin, Iran: Does woody plant encroachment follow global patterns?
Source: BMC Ecol Evol. 2026 Aug 17;26:72. doi: 10.1186/s12862-026-02575-z (PMC13488282; doi:10.1186/s12862-026-02575-z)
Supplement: Supplementary file 1 [file 12862_2026_2575_MOESM9_ESM.docx]

*Supplementary Information for*

**Plant community responses to climate change in the Zayandeh-Rud Basin, Iran: Does woody plant encroachment follow global patterns?**

**This file includes:**

**Table S1 - Table S6**

**Figure S1 - Figure S8**

Table S1. Variance Inflation Factor (VIF) of Environmental Variables

| **Environmental Variables** | **Variance Inflation Factor (VIF)** |
| --- | --- |
| Bio2 | 3.95 |
| Bio4 | 4.87 |
| Bio5 | 2.16 |
| Bio7 | 2.84 |
| Bio12 | 2.58 |
| Bio14 | 1.61 |
| Bio15 | 2.45 |
| Aspect | 1.22 |
| Slope | 2.08 |

|  | **bio1** | **bio2** | **bio3** | **bio4** | **bio5** | **bio6** | **bio7** | **bio8** | **bio9** | **bio10** | **bio11** | **bio12** | **bio13** | **bio14** | **bio15** | **bio16** | **bio17** | **bio18** | **bio19** | **dem** | **slope** | **aspect** |
| --- | --- | --- | --- | --- | --- | --- | --- | --- | --- | --- | --- | --- | --- | --- | --- | --- | --- | --- | --- | --- | --- | --- |
| **bio1** | 1 | .203** | .363** | 0.036 | .982** | .981** | -.134** | .951** | .996** | .996** | .995** | -.816** | -.844** | -.288** | -.223** | -.808** | -.868** | -.871** | -.698** | -.961** | -.546** | 0.001 |
| **bio2** | .203** | 1 | .910** | 0.036 | .355** | .078* | .757** | .128** | .186** | .202** | .193** | 0.008 | -0.009 | -.161** | .275** | 0.008 | -.141** | -.210** | .099** | -.135** | -.303** | -0.006 |
| **bio3** | .363** | .910** | 1 | -.312** | .465** | .299** | .418** | .293** | .322** | .334** | .387** | -.139** | -.163** | -.268** | .318** | -.141** | -.342** | -.417** | -0.018 | -.312** | -.410** | -.080* |
| **bio4** | 0.036 | 0.036 | -.312** | 1 | .103** | -.097** | .567** | 0.052 | .093** | .118** | -0.058 | -.167** | -.134** | .187** | -.467** | -.170** | .175** | .217** | -.242** | .064* | .070* | .216** |
| **bio5** | .982** | .355** | .465** | .103** | 1 | .939** | 0.039 | .918** | .983** | .983** | .970** | -.778** | -.810** | -.296** | -.201** | -.767** | -.849** | -.857** | -.656** | -.942** | -.575** | 0.008 |
| **bio6** | .981** | .078* | .299** | -.097** | .939** | 1 | -.308** | .940** | .973** | .967** | .990** | -.825** | -.856** | -.306** | -.227** | -.815** | -.889** | -.885** | -.711** | -.967** | -.543** | -0.031 |
| **bio7** | -.134** | .757** | .418** | .567** | 0.039 | -.308** | 1 | -.190** | -.108** | -.091** | -.193** | .243** | .245** | .072* | .103** | .244** | .236** | .202** | .250** | .203** | -0.012 | .113** |
| **bio8** | .951** | .128** | .293** | 0.052 | .918** | .940** | -.190** | 1 | .942** | .952** | .947** | -.858** | -.874** | -.241** | -.293** | -.853** | -.810** | -.798** | -.769** | -.890** | -.511** | 0.015 |
| **bio9** | .996** | .186** | .322** | .093** | .983** | .973** | -.108** | .942** | 1 | .996** | .986** | -.817** | -.849** | -.273** | -.262** | -.806** | -.865** | -.861** | -.705** | -.968** | -.549** | 0.016 |
| **bio10** | .996** | .202** | .334** | .118** | .983** | .967** | -.091** | .952** | .996** | 1 | .984** | -.834** | -.858** | -.271** | -.270** | -.827** | -.846** | -.845** | -.725** | -.945** | -.535** | 0.02 |
| **bio11** | .995** | .193** | .387** | -0.058 | .970** | .990** | -.193** | .947** | .986** | .984** | 1 | -.807** | -.838** | -.301** | -.191** | -.799** | -.882** | -.888** | -.684** | -.964** | -.549** | -0.018 |
| **bio12** | -.816** | 0.008 | -.139** | -.167** | -.778** | -.825** | .243** | -.858** | -.817** | -.834** | -.807** | 1 | .994** | .292** | .649** | .998** | .693** | .632** | .980** | .730** | .462** | -.091** |
| **bio13** | -.844** | -0.009 | -.163** | -.134** | -.810** | -.856** | .245** | -.874** | -.849** | -.858** | -.838** | .994** | 1 | .303** | .629** | .987** | .731** | .670** | .961** | .778** | .499** | -.085** |
| **bio14** | -.288** | -.161** | -.268** | .187** | -.296** | -.306** | .072* | -.241** | -.273** | -.271** | -.301** | .292** | .303** | 1 | -0.03 | .274** | .457** | .391** | .221** | .308** | .323** | -0.041 |
| **bio15** | -.223** | .275** | .318** | -.467** | -.201** | -.227** | .103** | -.293** | -.262** | -.270** | -.191** | .649** | .629** | -0.03 | 1 | .650** | 0.059 | -0.048 | .765** | .157** | .116** | -.219** |
| **bio16** | -.808** | 0.008 | -.141** | -.170** | -.767** | -.815** | .244** | -.853** | -.806** | -.827** | -.799** | .998** | .987** | .274** | .650** | 1 | .671** | .613** | .981** | .709** | .448** | -.083** |
| **bio17** | -.868** | -.141** | -.342** | .175** | -.849** | -.889** | .236** | -.810** | -.865** | -.846** | -.882** | .693** | .731** | .457** | 0.059 | .671** | 1 | .972** | .565** | .904** | .513** | -0.002 |
| **bio18** | -.871** | -.210** | -.417** | .217** | -.857** | -.885** | .202** | -.798** | -.861** | -.845** | -.888** | .632** | .670** | .391** | -0.048 | .613** | .972** | 1 | .486** | .902** | .505** | 0.029 |
| **bio19** | -.698** | .099** | -0.018 | -.242** | -.656** | -.711** | .250** | -.769** | -.705** | -.725** | -.684** | .980** | .961** | .221** | .765** | .981** | .565** | .486** | 1 | .601** | .382** | -.118** |
| **dem** | -.961** | -.135** | -.312** | .064* | -.942** | -.967** | .203** | -.890** | -.968** | -.945** | -.964** | .730** | .778** | .308** | .157** | .709** | .904** | .902** | .601** | 1 | .570** | 0.025 |
| **slope** | -.546** | -.303** | -.410** | .070* | -.575** | -.543** | -0.012 | -.511** | -.549** | -.535** | -.549** | .462** | .499** | .323** | .116** | .448** | .513** | .505** | .382** | .570** | 1 | 0.06 |
| **aspect** | 0.001 | -0.006 | -.080* | .216** | 0.008 | -0.031 | .113** | 0.015 | 0.016 | 0.02 | -0.018 | -.091** | -.085** | -0.041 | -.219** | -.083** | -0.002 | 0.029 | -.118** | 0.025 | 0.06 | 1 |

Table S2. Correlation matrix of Environmental Variab

Table S3. Threshold, Sensitivity, Specificity and Kappa (max-Kappa) for each community in study area

| **Community Type** | **Threshold** | **Sensitivity** | **Specificity** | **Kappa (max-Kappa)** |
| --- | --- | --- | --- | --- |
| Semi Steppe-Shrub | 0.34 | 0.96 | 0.88 | 0.76 |
| Semi Steppe-Semi Shrub | 0.15 | 0.98 | 0.80 | 0.52 |
| Semi Steppe-Perennial Herbaceous | 0.13 | 1.00 | 0.94 | 0.64 |
| Steppe-Semi Shrub | 0.24 | 0.80 | 0.89 | 0.38 |
| Alpine Vegetation | 0.12 | 0.96 | 0.95 | 0.82 |
| Semi Desert-Salty Plants | 0.17 | 0.98 | 0.88 | 0.59 |
| Semi Desert-Shrub | 0.16 | 0.98 | 0.95 | 0.60 |

Table S4. ODMAP v1.0 protocol

| **ODMAP section** | **ODMAP subsection** | **ODMAP elements** |
| --- | --- | --- |
| **Overview** | Model objective/model purpose | To (1) model the current spatial distribution of seven dominant plant communities in relation to key climatic and topographic variables; (2) identify the main environmental drivers shaping their distribution; (3) project changes in the spatial extent and patterns under mid-century (2050) and late-century (2070) climate scenarios (SSP1-2.6, SSP2-4.5, SSP5-8.5); and (4) compare projected changes in the distribution and suitable habitat of different woody plant communities under future climate scenarios to determine whether their responses are uniform or community-specific, and whether they collectively support the globally reported pattern of woody plant encroachment in dryland ecosystems. |
|  | Taxon | Focal taxon (plant communities) |
|  | Location | Iran- Zayandeh-Rud basin |
|  | Scale of analysis | Spatial extent (lon/lat), spatial resolution (1 km²), temporal extent (current, 2050, 2070) |
|  | Community data overview | Presence-only data derived from field surveys |
|  | Type of predictors | Climatic (WorldClim), topographic (slope, aspect), land use |
|  | Conceptual model/hypotheses | Climate variables influence plant community distribution |
|  | Assumptions | Static predictors (slope, aspect, land use) remain constant in future |
|  | SDM algorithms | GLM, BRT, RF, CART, SVM, MARS, RPART; MaxEnt ensemble modelling applied |
|  | Model workflow | Data preprocessing → variable selection → modelling → evaluation → ensemble → projection |
|  | Software | R (sdm package), GIS software, WorldClim |
| **Data** | Community data | Field-based occurrence data; sample size varies by community (45–346); presence-only; pseudo-absence generation applied |
|  | Data partitioning | Bootstrapping with random sampling with replacement was applied using 5 replicates. In each iteration, 70% of the data were randomly selected for model training and the remaining 30% were used for model testing. |
|  | Predictor variables | Selected bioclimatic and environmental variables; correlation and VIF filtering applied |
|  | Transfer data for projection | GCMs: MRI-ESM2-0, HadGEM3-GC31-LL; scenarios SSP1-2.6, SSP2-4.5, SSP5-8.5; years 2050, 2070 |
| **Model** | Variable pre-selection | Pearson correlation and VIF used to remove collinearity |
|  | Multicollinearity | Variables with VIF > 5 removed |
|  | Model settings/model complexity | Default and tuned settings applied depending on algorithm |
|  | Model estimates | Variable importance and response relationships assessed |
|  | Model selection/model averaging/ensembles | Ensemble modelling based on model performance metrics |
|  | Non-independence correction/analyses | Spatial autocorrelation not significant; no correction applied |
|  | Threshold selection | Optimal threshold selected using max(se+sp) (TSS criterion) |
| **Assessment** | Performance statistics | AUC, TSS, sensitivity, specificity, Cohen’s Kappa |
|  | Plausibility check | spatial prediction maps evaluated |
| **Prediction** | Prediction output | Habitat suitability maps; binary maps based on threshold |
|  | Uncertainty quantification | Variation across models, GCMs, and scenarios considered |
|  |  |  |

Table S5. **Mean ± SD of AUC and TSS values across five bootstrap replicates, separately for each algorithm**

| Models | GLM | BRT | RF | MaxEnt | CART | SVM | MARS | RPART |
| --- | --- | --- | --- | --- | --- | --- | --- | --- |
|  | Semi Steppe-Shrub | | | | | | | |
| AUC | **0.927 ± 0.011** | **0.959 ± 0.009** | **0.981 ± 0.004** | **0.958 ± 0.004** | **0.941 ± 0.016** | **0.955 ± 0.007** | **0.956 ± 0.009** | **0.935 ± 0.011** |
| TSS | **0.814 ± 0.041** | **0.819 ± 0.019** | **0.892 ± 0.022** | **0.825 ± 0.011** | **0.822 ± 0.026** | **0.814 ± 0.014** | **0.803 ± 0.031** | **0.818 ± 0.026** |
|  | Semi Steppe-Semi Shrub | | | | | | | |
| AUC | 0.850 ± 0.031 | 0.908 ± 0.029 | 0.948 ± 0.015 | 0.900 ± 0.029 | 0.854 ± 0.038 | 0.883 ± 0.024 | 0.884 ± 0.037 | 0.850 ± 0.041 |
| TSS | 0.577 ± 0.062 | 0.700 ± 0.082 | 0.788 ± 0.060 | 0.683 ± 0.039 | 0.632 ± 0.109 | 0.663 ± 0.064 | 0.680 ± 0.089 | 0.625 ± 0.079 |
|  | Semi Steppe-Perennial Herbaceous | | | | | | | |
| AUC | 0.865 ± 0.064 | 0.955 ± 0.022 | 0.978 ± 0.007 | 0.969 ± 0.011 | 0.847 ± 0.092 | 0.922 ± 0.041 | 0.908 ± 0.101 | 0.877 ± 0.104 |
| TSS | 0.674 ± 0.104 | 0.804 ± 0.084 | 0.861 ± 0.018 | 0.852 ± 0.020 | 0.676 ± 0.153 | 0.773 ± 0.087 | 0.775 ± 0.142 | 0.726 ± 0.177 |
|  | Steppe-Semi Shrub | | | | | | | |
| AUC | 0.753 ± 0.034 | 0.822 ± 0.026 | 0.871 ± 0.046 | 0.823 ± 0.020 | 0.768 ± 0.017 | 0.813 ± 0.025 | 0.808 ± 0.027 | 0.767 ± 0.008 |
| TSS | 0.446 ± 0.078 | 0.515 ± 0.050 | 0.599 ± 0.099 | 0.541 ± 0.049 | 0.439 ± 0.033 | 0.515 ± 0.035 | 0.519 ± 0.063 | 0.497 ± 0.035 |
|  | Alpine Vegetation | | | | | | | |
| AUC | 0.983 ± 0.007 | 0.988 ± 0.005 | 0.989 ± 0.010 | 0.984 ± 0.004 | 0.931 ± 0.067 | 0.966 ± 0.025 | 0.978 ± 0.014 | 0.934 ± 0.058 |
| TSS | 0.901 ± 0.029 | 0.938 ± 0.036 | 0.946 ± 0.031 | 0.907 ± 0.028 | 0.855 ± 0.117 | 0.881 ± 0.064 | 0.904 ± 0.037 | 0.850 ± 0.102 |
|  | Semi Desert-Salty Plants | | | | | | | |
| AUC | 0.929 ± 0.020 | 0.951 ± 0.012 | 0.972 ± 0.006 | 0.942 ± 0.013 | 0.877 ± 0.035 | 0.915 ± 0.013 | 0.932 ± 0.015 | 0.901 ± 0.023 |
| TSS | 0.775 ± 0.041 | 0.818 ± 0.054 | 0.854 ± 0.030 | 0.797 ± 0.054 | 0.734 ± 0.080 | 0.744 ± 0.040 | 0.798 ± 0.054 | 0.757 ± 0.054 |
|  | Semi Desert-Shrub | | | | | | | |
| AUC | 0.923 ± 0.034 | 0.965 ± 0.022 | 0.987 ± 0.008 | 0.968 ± 0.021 | 0.828 ± 0.100 | 0.962 ± 0.012 | 0.877 ± 0.171 | 0.887 ± 0.076 |
| TSS | 0.812 ± 0.061 | 0.857 ± 0.046 | 0.919 ± 0.043 | 0.887 ± 0.061 | 0.702 ± 0.152 | 0.839 ± 0.048 | 0.729 ± 0.337 | 0.763 ± 0.093 |

Table S6. Full projected changes in suitable habitats of plant communities under different GCMs (MRI-ESM2-0 and HadGEM3-GC31-LL) and SSP scenarios (SSP1-2.6, SSP2-4.5 and SSP5-8.5) for 2050 and 2070.

| **Plant Communities** | **Current area (km^2^)** | **MRI-ESM2-0** | | | | | | **HadGEM3-GC31-LL** | | | | | |
| --- | --- | --- | --- | --- | --- | --- | --- | --- | --- | --- | --- | --- | --- |
|  |  | **SSP1-2.6** | | **SSP2-4.5** | | **SSP5-8.5** | | **SSP1-2.6** | | **SSP2-4.5** | | **SSP5-8.5** | |
|  |  | **Loss %** | **Gain %** | **Loss %** | **Gain %** | **Loss %** | **Gain %** | **Loss %** | **Gain %** | **Loss %** | **Gain %** | **Loss %** | **Gain %** |
|  |  | **2050s** | | | | | | | | | | | |
| Semi Steppe-Shrub | 4644.65 | 18.33 | 11.33 | 19.77 | 5.62 | 48.71 | 5.16 | 14.92 | 12.13 | 28.74 | 10.82 | 37.95 | 21.53 |
| Semi Steppe-Semi Shrub | 6356.62 | 10.86 | 77.39 | 11.14 | 62.29 | 6.18 | 53.63 | 4.08 | 88.32 | 11.02 | 56.58 | 10.99 | 45.01 |
| Semi Steppe-Perennial Herbaceous | 2760.00 | 33.93 | 33.86 | 80.95 | 3.70 | 83.75 | 0.05 | 54.48 | 25.82 | 77.15 | 0.15 | 90.53 | 0.00 |
| Steppe-Semi Shrub | 18291.81 | 5.14 | 66.43 | 0.67 | 57.58 | 0.24 | 53.47 | 3.01 | 66.24 | 5.24 | 43.24 | 6.37 | 37.66 |
| Alpine Vegetation | 2574.46 | 39.19 | 8.79 | 49.55 | 0.64 | 51.92 | 14.01 | 47.92 | 3.84 | 55.82 | 13.22 | 58.81 | 15.49 |
| Semi Desert-Salty Plants | 5109.66 | 82.91 | 16.31 | 86.56 | 40.21 | 99.15 | 8.26 | 57.86 | 54.61 | 87.95 | 3.71 | 93.57 | 22.36 |
| Semi Desert-Shrub | 3091.67 | 51.13 | 38.47 | 63.20 | 48.84 | 83.90 | 21.35 | 61.40 | 36.22 | 61.66 | 15.04 | 82.75 | 10.14 |
|  |  | **2070s** | | | | | | | | | | | |
| Semi Steppe-Shrub | 4644.65 | 28.41 | 8.31 | 35.52 | 7.71 | 52.14 | 7.25 | 27.15 | 11.92 | 32.89 | 8.20 | 49.77 | 6.97 |
| Semi Steppe-Semi Shrub | 6356.62 | 4.57 | 64.48 | 9.33 | 44.91 | 5.55 | 35.20 | 14.62 | 70.45 | 12.96 | 27.96 | 14.49 | 22.43 |
| Semi Steppe-Perennial Herbaceous | 2760.00 | 68.11 | 7.10 | 95.83 | 0 | 96.62 | 0.03 | 63.33 | 4.54 | 82.99 | 0.10 | 99.03 | 0.00 |
| Steppe-Semi Shrub | 18291.81 | 0.08 | 56.34 | 3.18 | 51.07 | 0.68 | 47.65 | 1.94 | 40.77 | 2.27 | 39.88 | 11.09 | 36.23 |
| Alpine Vegetation | 2574.46 | 45.89 | 2.11 | 49.41 | 10.67 | 97.70 | 0 | 47.81 | 30.17 | 57.82 | 7.08 | 67.72 | 10.22 |
| Semi Desert-Salty Plants | 5109.66 | 71.89 | 46.80 | 84.80 | 50.43 | 99.99 | 2.21 | 72.57 | 28.95 | 97.49 | 3.59 | 99.86 | 12.55 |
| Semi Desert-Shrub | 3091.67 | 62.31 | 40.17 | 92.55 | 28.30 | 99.15 | 31.04 | 80.24 | 9.61 | 90.00 | 10.30 | 96.26 | 3.46 |

Fig. S1- Representative field photographs of the seven vegetation community types studied in the Zayandeh-Rud basin: (a) Semi Steppe-Shrub; (b) Semi Steppe-Semi Shrub; (c) Semi Steppe-Perennial Herbaceous; (d) Steppe-Semi Shrub; (e) Alpine Vegetation; (f) Semi Desert-Salty Plants; and (g) Semi Desert-Shrub

Fig. S2- Projected future spatial distribution of Semi Steppe-Shrub under SSP1-2.6, SSP2-4.5, and SSP5-8.5 scenarios in the MRI-ESM2-0 and HadGEM3-GC31-LL GCMs for 2050 and 2070

Fig. S3- Projected future spatial distribution of Semi Steppe-Semi Shrub under SSP1-2.6, SSP2-4.5, and SSP5-8.5 scenarios in the MRI-ESM2-0 and HadGEM3-GC31-LL GCMs for 2050 and 2070

Fig. S4- Projected future spatial distribution of Semi Steppe-Perennial Herbaceous under SSP1-2.6, SSP2-4.5, and SSP5-8.5 scenarios in the MRI-ESM2-0 and HadGEM3-GC31-LL GCMs for 2050 and 2070

Fig. S5- Projected future spatial distribution of Steppe-Semi Shrub under SSP1-2.6, SSP2-4.5, and SSP5-8.5 scenarios in the MRI-ESM2-0 and HadGEM3-GC31-LL GCMs for 2050 and 2070

Fig. S6- Projected future spatial distribution of Alpine Vegetation under SSP1-2.6, SSP2-4.5, and SSP5-8.5 scenarios in the MRI-ESM2-0 and HadGEM3-GC31-LL GCMs for 2050 and 2070

Fig. S7- Projected future spatial distribution of Semi Desert-Salty Plants under SSP1-2.6, SSP2-4.5, and SSP5-8.5 scenarios in the MRI-ESM2-0 and HadGEM3-GC31-LL GCMs for 2050 and 2070

Fig. S8- Projected future spatial distribution of Semi Desert-Shrub under SSP1-2.6, SSP2-4.5, and SSP5-8.5 scenarios in the MRI-ESM2-0 and HadGEM3-GC31-LL GCMs for 2050 and 2070
